# Acute Angle Closure Glaucoma in a Patient With Jalili-Smith Syndrome

**DOI:** 10.7759/cureus.68670

**Published:** 2024-09-04

**Authors:** Sruthi Suresh, Hafsa Z Zuberi, Rahul Khandekar, Emily B Buchanan, Karanjit S Kooner

**Affiliations:** 1 Ophthalmology, University of Texas Southwestern Medical Center, Dallas, USA; 2 School of Medicine, Texas Tech University Health Sciences Center, Lubbock, USA; 3 Ophthalmology, University of Texas Health Science Center at San Antonio, San Antonio, USA

**Keywords:** cone-rod dystrophy, oct (optical coherence tomography), optical coherence tomography angiography, glaucoma, jalili-smith syndrome

## Abstract

We describe a 29-year-old Iranian male with Jalili-Smith syndrome (JSS), who presented with acute angle closure glaucoma. JSS is a rare autosomal recessive oculo-dental disorder characterized by cone-rod dystrophy and amelogenesis imperfecta. Though the disease is observed worldwide, many cases are concentrated in the Gaza Strip. Consanguinity is an important risk factor. Patients typically present with photophobia, nystagmus, and enamel deformation. Our patient exhibited nystagmus, photophobia, cataracts, hyperopia, narrow-angle glaucoma, marked thinning of the retina, and bull’s eye maculopathy. In addition, we describe the findings of optical coherence tomography angiography (OCTA). Our patient also underwent phacoemulsification in both eyes with concomitant minimally invasive glaucoma surgeries (MIGS). To the best of our knowledge, narrow-angle glaucoma, OCTA findings, and cataract surgery combined with MIGS have not been reported before in patients with JSS.

## Introduction

Jalili-Smith syndrome (JSS) is a rare oculo-dental disorder of autosomal recessive inheritance that is characterized by a combination of cone-rod dystrophy (CRD) and amelogenesis imperfecta (AI) [1]. Most cases are reported in consanguineous families in the Eastern Mediterranean, Africa, and Kosovo [2]. The disorder was first described in 29 members of an extended Arab family in the Gaza Strip in 1988 [1]. The syndrome is believed to be caused by mutations of the cyclin and cystathionine-β-synthase (CBS) domain divalent CNNM4 (metal cation transport mediator 4) gene [3]. In the Gaza Strip, 83% of patients with CRDs had JSS [4]. Ocular manifestations of JSS include CRDs, loss of central vision, dyschromatopsia, and photophobia in the second and third decades of life.

## Case presentation

In 2017, a 29-year-old Iranian male diagnosed with JSS in early childhood with prior confirmatory genetic testing (c.1091delGly(p.364Valfs*10)) was seen in our clinic with an acute angle closure glaucoma in the left eye (OS). The intraocular pressures (IOPs) measured were 18 mm Hg in the right eye (OD) and 36 mm Hg in the OS. His visual acuity was 20/400 in both eyes (OU), and he complained of photophobia and pain. The slit-lamp examination revealed shallow chambers and cataractous changes in OU. The anterior chamber (AC) depths were 2.19 mm and 1.63 mm, respectively. Lens thickness measured by biometry was 4.34 mm and 4.28 mm and the axial lengths were 23.42 mm and 24.07 mm (LENSTAR, Haag-Streit Diagnostics USA, Mason, OH).

The indirect gonioscopy revealed narrow angles open to the anterior trabecular meshwork. With dynamic gonioscopy (indentation), the scleral spur was partially visible in some areas but no ciliary body band was seen. The iris base covered most of the scleral spur. We also noticed a double hump sign indicating a plateau iris.

The left pupil was mid-dilated and fixed (atonic pupil). The central corneal thickness (CCT) values (μ) were 594 and 596. The refraction was +4.5 diopters OU. The cup-to-disk ratios (CDRs) were 0.35 OD and 0.45 OS. The fundus examination showed evidence of hypoplastic optic discs and pallor OU, with persistent glial tissue in the optic cup OD. The elevated IOPs were successfully lowered using apraclonidine 1%, brimonidine 0.2%, dorzolamide hydrochloride 2% - timolol maleate 0.5 %, latanoprost 0.005%, and pilocarpine 2% to 8 mm Hg OD and 22 mm Hg OS.

**Figure 1:**
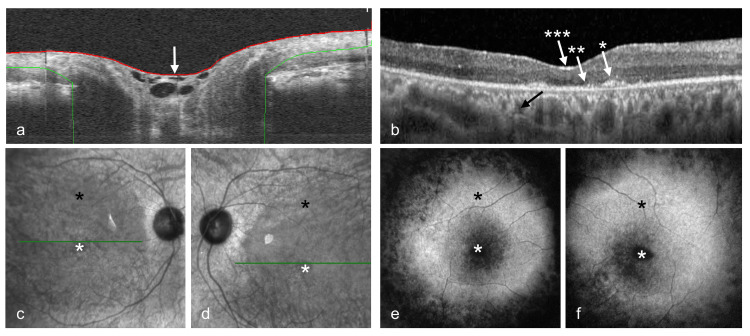
Optical coherence tomography and fundus photography findings. (a) B-scan of the optic disk right eye (OD) showing persistent glial tissue bridging the optic cup (white arrow). (b) B-scan showing the presence of epiretinal membranes (arrow with three asterisks), rarefaction, structural disorganization of the external limiting membrane and ellipsoidal zone (arrow with one asterisk), and drusen-lipofuscin complex (arrow with two asterisks), and prominent choroidal vasculature (black arrow) OD. (c, d) Red-free photographs depicting the presence of bull’s eye maculopathy with a central round dark center (white asterisk) surrounded by round hypopigmentation (black asterisk) OD and left eye (OS), respectively. (e, f) Higher magnification view of the bull’s eye maculopathy OD and OS, respectively.

Spectralis optical coherence tomography (OCT) B-scans (Heidelberg Engineering®, Heidelberg, Germany) revealed hypoplastic optic disks OU with persistent glial tissue bridging the optic cup OD (Figure 1A). The retinal scans showed disruption of the ellipsoid zone (EZ), interdigitation zone (IZ), epiretinal membranes, lipofuscin-drusen complex, and rarefaction (Figure 1B). The underlying choroidal vasculature was highly visible due to atrophy of the retinal pigment epithelium (RPE) (Figures 1B, 1C). Red-free imaging showed the bull’s eye maculopathy manifesting a round dark center encircled by hypopigmented halos OU (Figures 1C-1F).

**Figure 2:**
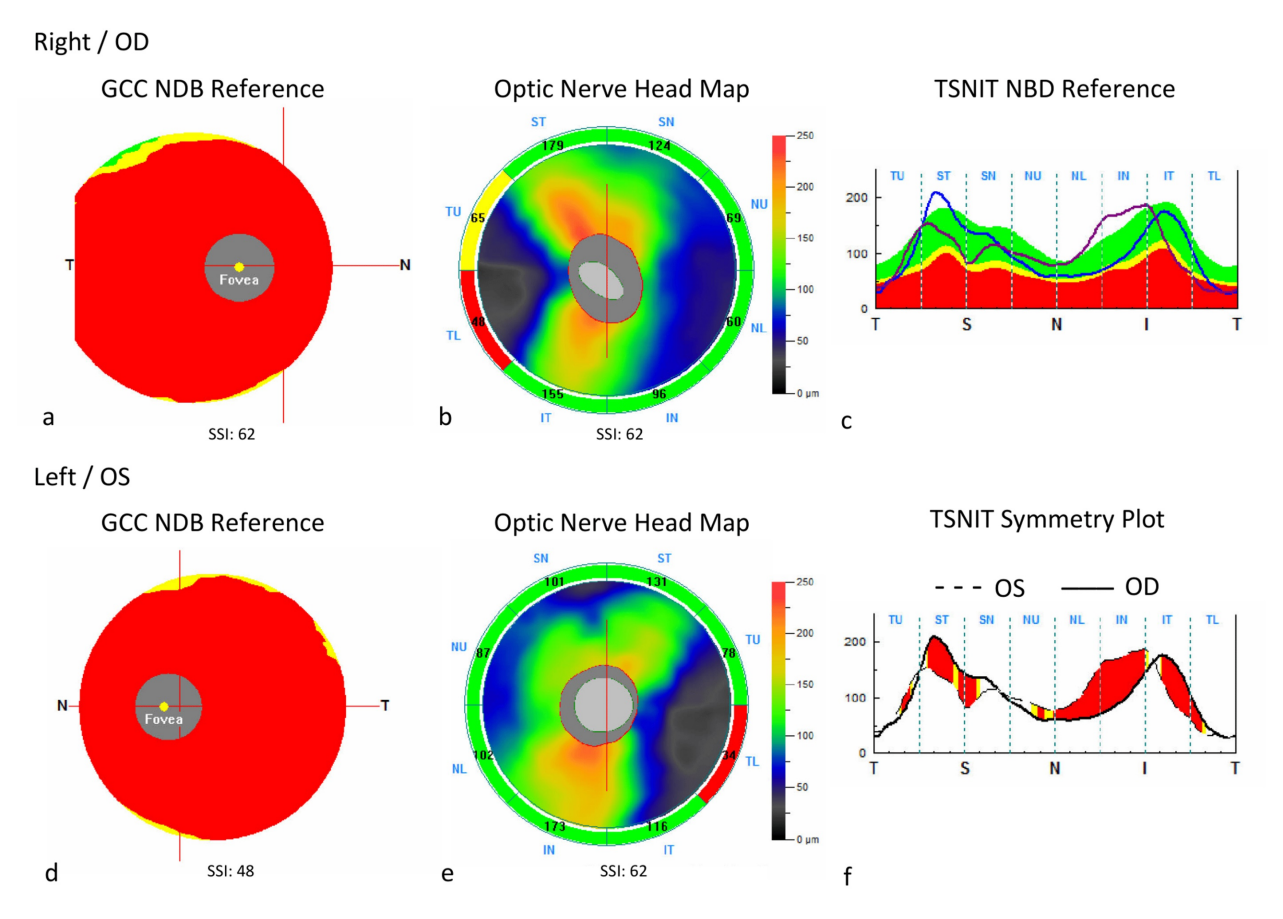
Optic nerve head/ganglion cell complex report with corresponding heatmaps of OD (a-b) and OS (d-e). Marked thinning of the GCC layer is depicted in red (a, d), and normal nerve fiber layer thickness is depicted in green (b, e) compared with normative database (NDB) illustrations. Corresponding plots of normal retinal nerve fiber layer thickness in the temporal-superior-nasal-inferior-temporal (TSNIT) quadrants are also shown (c, f). OD: right eye; OS: left eye; GCC: ganglion cell complex.

Optical coherence tomography angiography (OCTA) imaging utilizing Optovue AvantiXR AngioVueHD software version A2018.0.0.18 scanner (Optovue®, Freemont, CA) was also performed. Retinal nerve fiber layer (RNFL) values were normal (Figure 2) OU: average RNFL (100 µm and 103 µm); superior RNFL thickness (109 µm and 99 µm); inferior RNFL thickness (90 µm and 106 µm). However, the ganglion cell complex (GCC) analysis showed marked thinning, and values were as follows: average GCC thickness (43 µm and 49 µm); superior GCC thickness (43 µm and 49 µm); inferior GCC thickness (43 µm and 50 µm). In addition, focal loss volume (FLV), defined as the area of focal GCC loss (24.53% and 13.29%), and global loss volume (GLV), defined as gross thinning of GCC (53.62% and 46.99%), were both markedly increased.

**Figure 3:**
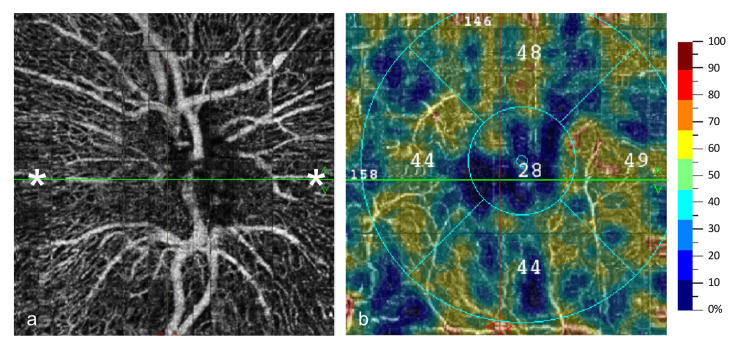
Optical coherence tomography angiography findings. (a) En-face OCTA scan of the retina OD showing areas of normal radial peripapillary capillaries (white asterisks). (b) En-face OCTA scan of the optic disk OD depicting normal macular vessel density. OD: right eye; OCTA: optical coherence tomography angiography.

The radial peripapillary capillaries were normal (Figure 3A), and macular vessel density OD was normal (46.25%, Figure 3B).

As the AC depth and gonioscopic features stayed unchanged, we performed laser peripheral iridotomy (LPI) OU. AC depth and angle features stayed unchanged after LPI but the IOPs were stable, being 14 mmHg OU. In 2018, the patient showed progressing cataracts OU. We treated it by performing cataract surgery and an iStent® (Glaukos Corp., San Clemente, CA) OS followed by cataract surgery with Kahook Dual Blade® (New World Medical Inc., Rancho Cucamonga, CA) goniotomy OD in 2019. At the last follow-up visit in June 2021, his visual acuity was 20/400 OU and IOPs were 9 mmHg OD and 8 mmHg in the OS. The postoperative gonioscopic features were unchanged. The iStent was in place OS, and OD showed a linear trabecular window devoid of the meshwork. The postoperative AC depths (mm) were 2.75/2.21, respectively. His current medications consist of brimonidine 0.2% OS twice per day (BID), Cosopt® 0.5% OU BID, and latanoprost 0.005% OU at bedtime.

In summary, besides the ocular components as described above, our patient also had a strong family history of JSS. He is a member of a large consanguineous Iranian family affected by JSS. The family was enrolled in a 2016 study conducted by Rahimi-Aliabadi et al. They described ocular and dental features in four members, but our patient was not in the study [5]. Within the patient’s family, there was a wide range of ocular features, including nystagmus, photophobia, poor vision, color blindness, macular coloboma, waxy discs and optic atrophy, and flat or distinguished electroretinogram and electrooculogram [5].

## Discussion

We describe a 29-year-old Iranian male with a combination of classic JSS features and signs and symptoms of acute angle closure glaucoma. The latter was initially treated medically followed by laser iridotomy. The OCTA imaging findings included normal RNFL, hypoplastic discs, normal peripapillary, and retinal capillary densities, but markedly reduced GCC thickness, thinning of both FLV and GLV, retinal degeneration (bony spicules), and bull’s eye retinopathy. The marked disconnect between GCC, RNFL, and vessel densities of both the retina and optic nerve is an important finding in our patient and has not been previously reported. We believe that without the OCTA technology, these unique findings may not have become apparent. It is also interesting that our findings correlate the CRD with the thinning/atrophy of GCC but not the RNFL or vessel densities. Therefore, we believe that both the compromised photoreceptors and GCC may explain poor vision in these patients because of the partial transmission of electrical information.

The presence of shallow chambers, anatomical narrow iridocorneal angles, and features of plateau iris, but the normal thickness of the crystalline lenses are also unique features in our patient. Dynamic gonioscopy revealed that the iris base was attached more anteriorly covering most of the scleral spur as only a small strip of it was visible in some areas. We also noticed a double-hump sign indicating plateau iris. All of these features combined explain why our patient developed acute angle closure glaucoma and responded well to laser iridotomy OU. Later, as the patient developed cataracts, we removed them using phacoemulsification, intraocular lens insertion, and minimally invasive glaucoma surgery (MIGS; iStent® and Kahook Dual Blade®). We believe that the anterior segment features would have been more apparent if we had access to ultrasound biomicroscopy (UBM).

Finally, we also describe OCTA imaging results including normal RNFL, thinning of GCC, disruption of EZ, normal foveal vessel density, and normal peripapillary capillaries. Previous studies have only mentioned macular thinning, macular volume loss, retinal atrophy, and loss of EZ in the fovea [5-7]. There is a wide variability in the clinical presentation of patients with JSS, as described in Table 1.

**Table 1:** Ocular features in patients with Jalili-Smith syndrome. Abbreviations: AI: amelogenesis imperfecta; F: female; HM: hand motion; OCT: optical coherence tomography; OCTA: optical coherence tomography angiography; M: male; N: no; NLP: no light perception; Y: yes.

Study, year	Sample size (gender)	Age range (year)	Origin; family history (Y/N)	Visual acuity	Fundus	Nystagmus (Y/N)	Ocular features	OCT/OCTA findings	Systemic features
Jalili and Smith, 1988 [1]	29 (13F - 16M)	25 - 50	Palestine; Y	20/120 to NLP	Bull’s eye maculopathy, bony spicules, optic nerve atrophy	Y	Achromatopsia	N/A	Hypocalcified AI variant: rough tooth surfaces, absent enamel layer
Jalili, 2010 [4]	3 (2F - 1M)	5 - 10	Palestine; Y	20/600 - 20/200	Minor retinal epithelial defects	Y	Achromatopsia, hypermetropia	N/A	AI associated with calculus formation
Rahimi-Aliabadi et al., 2016 [5]	4 (2F - 2M)	25 - 39	Iran; Y	HM - 20/150	Macular coloboma, bull’s eye maculopathy, optic atrophy	Y	Photophobia, achromatopsia	OCT: Macular thinning	AI
Hirji, 2018 [7]	7 (1F - 6M)	3 - 16	Kosovo, Pakistan, England/Ireland/Germany, Afghanistan; Y	20/98 - 20/480	Macular and retinal atrophy	Y	Photophobia, optic disc pallor, no rod and cone responses	OCT: Severe outer retinal atrophy	Hypoplastic AI variant, enamel hypoplasia
Prasov, 2020 [6]	3 (1F - 2M)	3 - 16	North and Central America; N	20/160 - 20/360	Bull's eye maculopathy, retinal atrophy	2; Y 1; N	Photophobia	OCT: Loss of ellipsoid and interdigitation zone in the fovea	Hypoplastic AI, one patient had spastic paraplegia and fatty liver
Current study, 2024	1 (1M)	29	Iran; Y	20/400	Disc pallor, bony spicules	Y	Narrow-angle glaucoma, cataracts, hyperopia, photophobia, hypoplastic discs	OCT: Loss of ellipsoid and interdigitation zone in the fovea, bull’s eye maculopathy, GCC thinning. OCTA: Normal vessel density in the macula	AI with artificial dentition

## Conclusions

We believe our case report is the first to describe acute angle glaucoma, OCTA findings, and cataract removal combined with MIGS in a patient with JSS. We recommend close collaboration between glaucoma, retina specialists, and geneticists to effectively manage patients with JSS.
